# The endoribonuclease N4BP1 prevents psoriasis by controlling both keratinocytes proliferation and neutrophil infiltration

**DOI:** 10.1038/s41419-021-03774-w

**Published:** 2021-05-14

**Authors:** Chenliang Gou, Wenkai Ni, Panpan Ma, Fengbo Zhao, Zhou Wang, Rong Sun, Yingcheng Wu, Yuanyuan Wu, Miaomiao Chen, Hao Chen, Jie Zhang, Yu Shen, Mingbing Xiao, Cuihua Lu, Renfang Mao, Yihui Fan

**Affiliations:** 1 Department of Pathogenic Biology, School of Medicine, Nantong University, 226001 Jiangsu, China; 2 Department of Immunology, School of Medicine, Nantong University, 226001 Jiangsu, China; 3 Department of Gastroenterology, Affiliated Hospital of Nantong University, 226001 Jiangsu, China; 4 Laboratory of Medical Science, School of Medicine, Nantong University, 226001 Jiangsu, China; 5 Department of Pathophysiology, School of Medicine, Nantong University, 226001 Jiangsu, China; 6 School of Life Sciences, Nantong University, 226001 Jiangsu, China; 7 Department of dermatology, The third Affiliated Hospital of Nantong University, Jiangsu, China

**Keywords:** Immunology, Psoriasis

## Abstract

Psoriasis is a common chronic skin disease, characterized by abnormal interplay between hyperproliferative epidermal keratinocytes and self-reactive immune cells with not fully addressed molecular mechanism. N4BP1 (NEDD4-binding protein 1) is considered as an immune regulator for a long time but its physiological role is not determined yet. Here, we found that the expression of N4BP1 in skin was highest among all 54 tested tissues, and its expression was further upregulated in psoriatic skin. N4BP1-deficient mice exhibited normal grossly, but developed severe and prolonged IMQ-induced psoriasis-like disease comparing to controls. N4BP1 mainly expressed in keratinocytes and located on nucleus. Up- but not downregulated genes in N4BP1-deficient skin were specifically enriched in keratinocyte proliferation and differentiation. The proliferation of N4BP1-deficient primary keratinocytes was faster compared to that of controls. The upregulated genes upon ablation of N4BP1 were highly enriched in targets of AP-1 transcription factor. Knocking out N4BP1 resulted in upregulation of JunB and FosB, and conversely, overexpression of N4BP1 greatly reduced their expression. Furthermore, N4BP1 binds with JunB and FosB encoding mRNAs and greatly reduces their stability. In addition, with a high expression in neutrophils, N4BP1 limits survival of neutrophils in blood and infiltration of neutrophils in psoriatic skin by targeting CXCL1, CCL20, and S100A8. These findings demonstrate that N4BP1 controls the proper function of keratinocytes and neutrophils by negatively regulating JunB, FosB, and CXCL1, respectively, and that is critical for psoriasis prevention.

## Introduction

Psoriasis, affecting millions of people worldwide, is chronic localized or systemic skin disease characterized by areas of red, inflamed, itchy, thickened skin, and often covered with silvery scales^1,2^. The cellular hallmark of psoriasis is sustained T cell-, neutrophil-, dendritic cell-, and macrophage-triggered inflammation that leads to uncontrolled keratinocyte proliferation and dysfunctional differentiation^1,2^. The abnormal and mutually activated crosstalk between immune cells and keratinocytes is the central pathologic process for psoriasis, but the underlying molecular mechanism and the initial factors are still not fully understood^3,4^. Among the identified abnormal molecular networks, TNFa-, IL-23-, and IL-17-associated signaling are dominant and all of them became therapeutic targets in clinic^5–8^. However, even though the tremendous progress achieved, psoriasis still cannot be cured but can be controlled with continuous medication. Thus, further dissecting the molecular mechanism is critical to fully understand psoriasis and achieve a cure.

Keratinocytes is the major component of the epidermis and its abnormal proliferation and differentiation is the character of psoriasis^9^. Keratinocytes play critical roles throughout the whole pathologic process of psoriasis from initiation, maintenance to relapse^10^. Initially, keratinocytes were considered as a main driver of psoriasis and inhibiting proliferation of keratinocytes by methotrexate is effective for severe, disabling psoriasis^9,10^. Afterwards, immune cells especially T cells and their produced inflammatory cytokines were observed and biologics targeting on these inflammatory cytokines have been developed for therapy in clinic^11^. From that, keratinocytes in skin are more considered as an executor to display the function of immune cells. However, recent investigations by genetic modification in keratinocytes provide several new evidence to rethink the intrinsically driving role of keratinocytes in psoriasis. For example, keratinocyte-specific deletion of TNIP1 promotes IL-17- and IMQ-induced psoriasis^12^. Genetic deletion of JunB and JunC in keratinocytes results in chemokine and cytokine overproduction and sufficiently trigger psoriasis in mice^13,14^. Mice with specific deletion of IKK2 in keratinocytes developed psoriasis-like plaques due to uncontrolled TNFR1 signaling-mediated generation of IL-24^15^. These studies clearly demonstrate that abnormal keratinocyte might be sufficient to trigger psoriasis even with normal immune cells. Therefore, it is still under debate about whether abnormal keratinocytes or immune cells trigger psoriasis. The answer might be case-by-case but definitely important to prevent or cure psoriasis in future.

Neutrophils, the most abundant leukocytes in the circulation with a very short half-life of ~8 h, is one of the major histopathological hallmarks of psoriasis^16–18^. A pooled study including a large number of patients showed significant higher neutrophil-to-lymphocyte ratio in patients with psoriasis compared to it in controls^19^. Depletion of neutrophils with anti–Ly6G Ab greatly limits the development of imiquimod (IMQ)-induced psoriatic lesions in mice^20^. Accumulation of neutrophils in psoriasic skin forming Munro’s microabscesses was described a century ago^21^. Although neutrophils were documented in psoriasis as early as psoriasis was reported, the critical role of neutrophils in psoriasis is largely undermined. Furthermore, the molecular regulation of neutrophil maturation and survival is poorly explored.

Endoribonucleases are involved in virtually all general processes associated with eukaryotic RNA metabolism, and several sequence-specific endoribonucleases play essential roles in immune hemostasis through controlling quantitative changes of selected transcripts^22,23^. The Zc3h12a-like NYN domain subfamily of endoribonucleases includes seven members: ZC3H12A (Regnase-1), ZC3H12B, ZC3H12C, ZC3H12D, KHNYN, NYNRIN, and N4BP1(NEDD4-binding partner-1)^24,25^. Regnase-1 is a cytoplasmic endoribonuclease and plays essential role in controlling immune hemostasis^26^. Germline deletion of Regnase-1 in mice show growth retardation with severe splenomegaly and lymphadenopathy, and majority of mice die within 12 weeks of birth. Conditional deletion of Regnase-1 in bone marrow cells also results in severe inflammation involved in multiple organs^27,28^. Regnase-1 is upregulated in psoriatic skin and genetic knockout of Regnase-1 in keratinocyte disrupts skin integrity and promotes skin inflammation^29,30^. These phenotypes demonstrate that Regnase-1 has nonredundant critical roles in restricting inflammation. N4BP1 is originally identified as a NEDD4-binding protein to inhibit the E3 ligase Itch^31,32^. A recent work shows that N4BP1 inhibits HIV-1 replication by directly interacting and degrading viral mRNA in cells^33^. However, the physiological function of N4BP1 and its role in skin inflammation is unknown. Here, we found that N4BP1 plays critical role both in keratinocytes and neutrophils through specifically regulating mRNA targets including JunB, FosB, and CXCL1.

## Results

### The expression of N4BP1 is dominant in skin and further upregulated during psoriasis

**Fig. 1:**
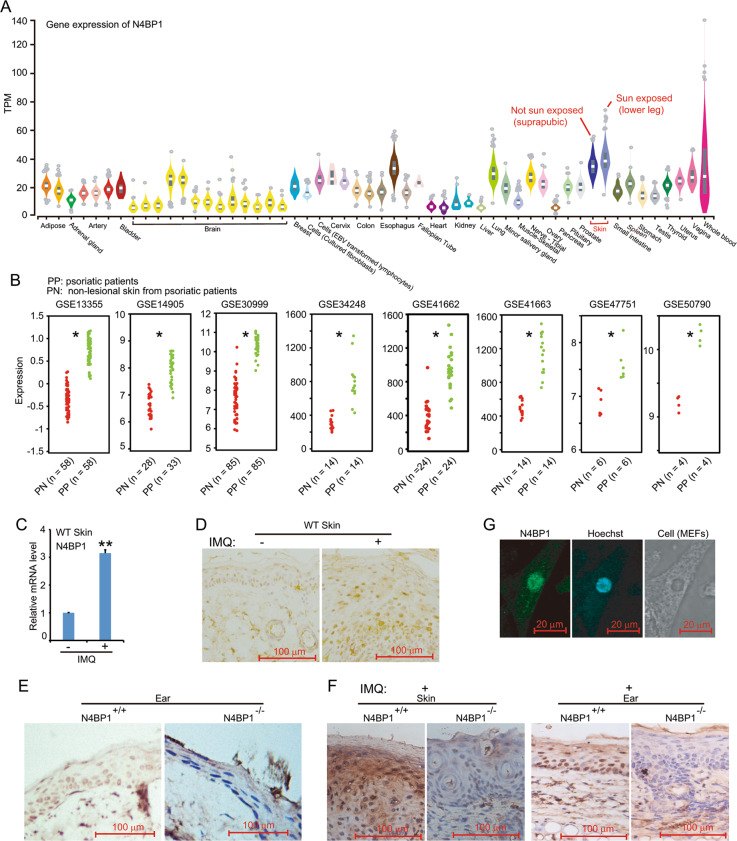
The characteristics of N4BP1 in skin and patients with psoriasis. **A** The raw data (Version 7) of RNA-seq from Genotype-Tissue Expression (GTEx) program was downloaded and reanalyzed (https://gtexportal.org/home). The relative expression of N4BP1 in 54 human tissues was present. **B** The raw data of RNA array were downloaded from following GEO datasets (GSE13355, GSE14905, GSE30999, GSE34248, GSE41662, GSE41663, GSE47751, and GSE50790). Both RNA array and RNA-seq data were reanalyzed. The expression of N4BP1 in lesioned and non-lesioned skin from each psoriatic patient was demonstrated as each dot. **C** The expression of N4BP1 in normal and IMQ-induced psoriatic skin from back of mice was examined by real-time RT-PCR. **D** The expression of N4BP1 in normal and IMQ-induced psoriatic skin from back of mice was examined by immunohistochemistry. **E** The expression of N4BP1 was examined by immunohistochemistry using anti-N4BP1 antibodies in N4BP1 wild-type and knockout mice from ear skin. **F** The expression of N4BP1 in IMQ-induced psoriatic skin from back and ear was examined by immunohistochemistry in N4BP1 wild-type and knockout mice. **G** The intracellular distribution of N4BP1 was determined by immunofluorescence in mouse embryonic fibroblasts (MEFs). The images (**D, E, F, G**) show representative data from one of three independent experiments. Data (**C**) from one of three experiments are shown. *P < 0.05; **P < 0.01.

Unlike Regnase-1, the function and characteristic of N4BP1 was largely unknown. To systematically assess N4BP1, we explored the expression of N4BP1 using dataset generated through Genotype-Tissue Expression (GTEx) program. The newest data (V7) including ~11,688 RNA-seq samples across 54 tissues with adequate power to truly demonstrate the gene expression across different tissues (https://gtexportal.org/home). In all analyzed tissues, N4BP1 shows a highest expression in skin (Fig. 1A). It is interesting that the expression of N4BP1 is further upregulated in sun-exposed skin (lower leg) compared that of not sun-exposed skin (suprapubic) (Fig. 1A). These results demonstrate that N4BP1 has dominant expression in skin, suggesting a potential important role of N4BP1 in skin. RNA sequencing of psoriatic skin and non-lesional skin from psoriatic patients shows increased expression of N4BP1 in psoriatic patient, and the enhanced expression of N4BP1 in psoriatic skin is highly consistent and observed in all reanalyzed GEO datasets (Fig. 1B). To further examine the expression of N4BP1, we established the imiquimod (IMQ)-induced psoriasis in mice. The mRNA level of N4BP1 was significantly upregulated in IMQ-induced skin compared to controls (Fig. 1C). Furthermore, immunohistochemistry analysis also shows the upregulated N4BP1 protein level in IMQ-induced skin (Fig. 1D). N4BP1 mainly located in the nucleus of keratinocytes (Fig. 1D–F). The specificity of anti-N4BP1 antibody in immunohistochemistry analysis was confirmed by using N4BP1^+/+^ and N4BP1^−/−^ skin both in normal and IMQ-stimulating condition (Fig. 1E and F). To further confirm the nuclear distribution of N4BP1, we isolated mouse embryonic fibroblasts (MEFs) and performed immune fluorescence assay to detect the location of N4BP1 in MEFs. Consistently, the results clearly show that N4BP1 mainly locates at nucleus (Fig. 1G). Taken together, our results demonstrate that N4BP1 highly expresses in skin and mainly expresses in keratinocytes with a nuclear distribution, and the expression of N4BP1 is further upregulated in psoriatic skin.

### N4BP1-deficient mice develop severe and prolonged IMQ-induced psoriasis-like disease

To uncover the physiological role of N4BP1, we established N4BP1-deficient mouse through CRISPR-Cas9-mediated deletion of exon 2 (Fig. S1A). Deletion of exon 2 results in a frameshift mutation that leads to an early translational termination. The generation of mutated allele was confirmed by polymerase chain reaction (Fig. S1B) and sequencing (data not show). By western blot and immunohistochemistry analysis, there is no detectable N4BP1 protein in skin from N4BP1^−/−^ mice (KO), through it can be easily detected in skin from N4BP1^+/+^ mice (WT) (Figs. 1E, F, 4E and data not shown). Breeding of N4BP1^+/−^ male and female mice gave rise offsprings with wild-type (WT), heterozygote (HET), and knockout (KO) genotypes in a Mendelian ratio, suggesting that N4BP1^−/−^ mice are viable (Data not shown). N4BP1^−/−^ mice are fertile and no apparent morphological defects compared to control mice when housed in pathogen-free condition.

**Fig. 2:**
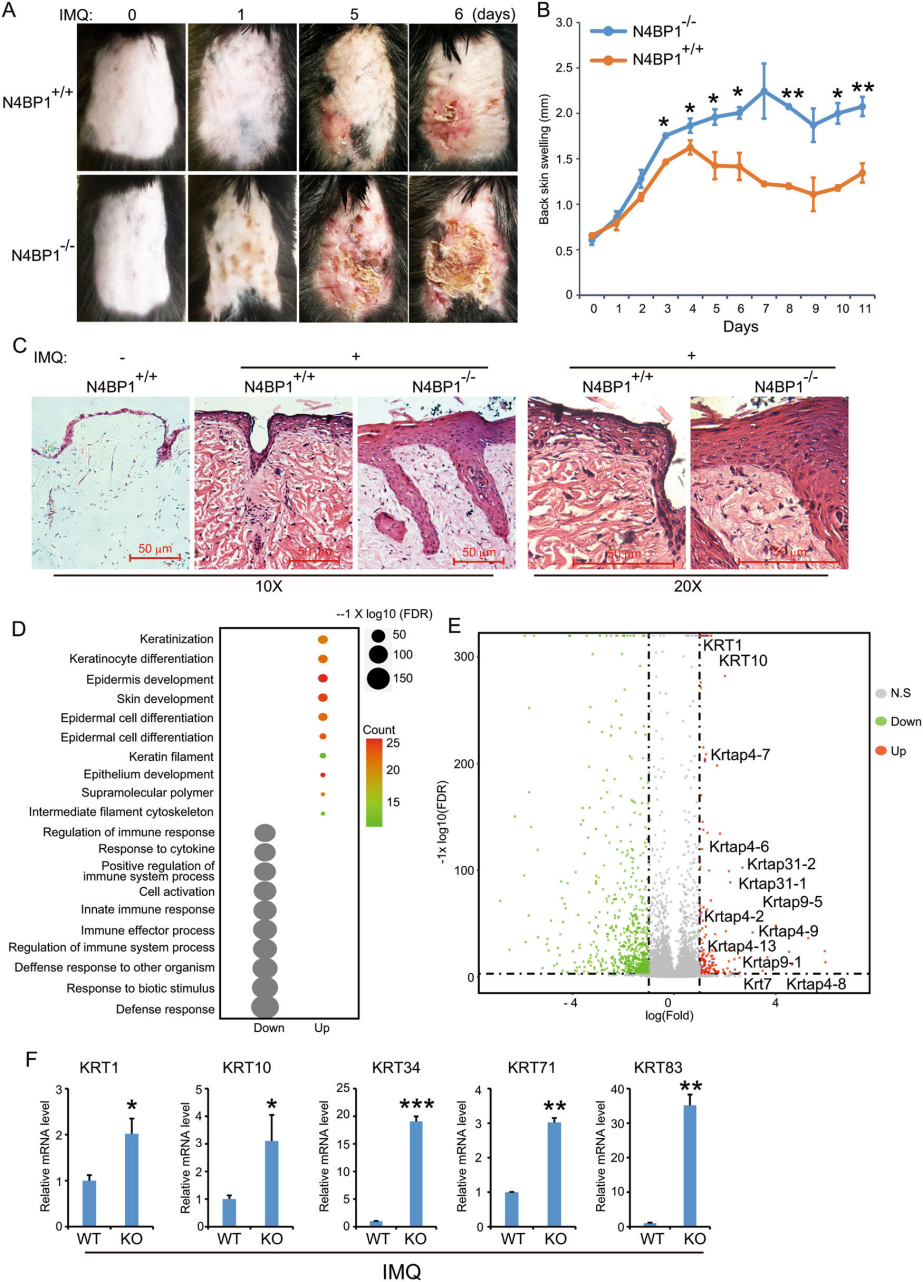
N4BP1-deficient mice develop severe and prolonged IMQ-induced psoriasis-like disease. **A** Representative photos of IMQ-induced psoriatic skin of N4BP1 wild-type and knockout mice. **B** Thickness of back skin from lesioned location of N4BP1 wild-type and knockout mice. **C** Hematoxylin and eosin staining of representative skin sections from indicated each group. **D** The most profoundly up- or downregulated genes in IMQ-treated N4BP1 knockout mice were subjected to analyze the enrichment of GO biological process by using online GSEA (geneset enrichment analysis) program. The enriched GO biological processes was shown. **E** Volcano plot was used to present the results of RNA-seq from IMQ-treated back skin between N4BP1 wild-type and knockout mice. The most-upregulated genes encoding keratins and keratin-associated proteins were labeled. **F** The upregulated genes encoding keratins including KRT1, KRT10, KRT34, KRT71, and KRT83 were further examined in IMQ-treated skin from N4BP1 wild-type and knockout mice by real-time RT-PCR analysis. The images show representative data from one of three independent experiments. The images (**A, C**) show representative data from one of three independent experiments. Data (**B, F**) from one of three experiments are shown. Statistical differences between groups were determined by the Student’s *t*-test. *P < 0.05; **P < 0.01; ***P < 0.001.

Our study on the expression of N4BP1 showed that N4BP1 has a highly expression in skin and indicated a function in psoriasis. To assess whether N4BP1 plays a role in psoriasis, we treated the back skin of WT and KO mice with IMQ, a common medicine used to induce psoriasis in mice. As shown in Fig. 2A, the back skin from KO mice display more erythema and scaling compared to that from WT mice. Consistently, IMQ induces more swelling of back skin in KO mice comparing to that in WT mice back skin (Fig. 2B). Next, we performed immunohistochemistry in KO and WT mice to further analyze the pathological changes. Consistent with a role of N4BP1 in psoriasis, the hyperkeratosis, parakeratosis and acanthosis were more pronounced in KO mice compared to that in WT control mice (Fig. 2C). We got similar results in the skin from ears by treating with IMQ in WT and KO mice (Fig. S1C). To further explore our finding in molecular level, we did RNA sequencing of IMQ-treated skin from WT and KO mice. 171 profoundly upregulated genes were identified from KO mice and control pairs (Expression > 1, FDR < 0.001, and Fold > 2). Consistent with severe IMQ-induced psoriasis in KO mice, the upregulated genes are significantly enriched in psoriasis-associated process, such as keratinization, keratinocyte differentiation, and epidermis development (Fig. 2D). A list of genes encoding keratins (KRTs) and keratin-associated proteins (KRTAPs) are significantly upregulated in KO mice (Fig. 2E). The upregulation of KRT1, KRT10, KRT34, KRT71, and KRT83 in KO mice was further confirmed by RT-PCR (Fig. 2F). Collectively, N4BP1 negatively regulates IMQ-induced psoriasis in vivo.

### Loss of N4BP1 results in increased keratinocyte proliferation

**Fig. 3:**
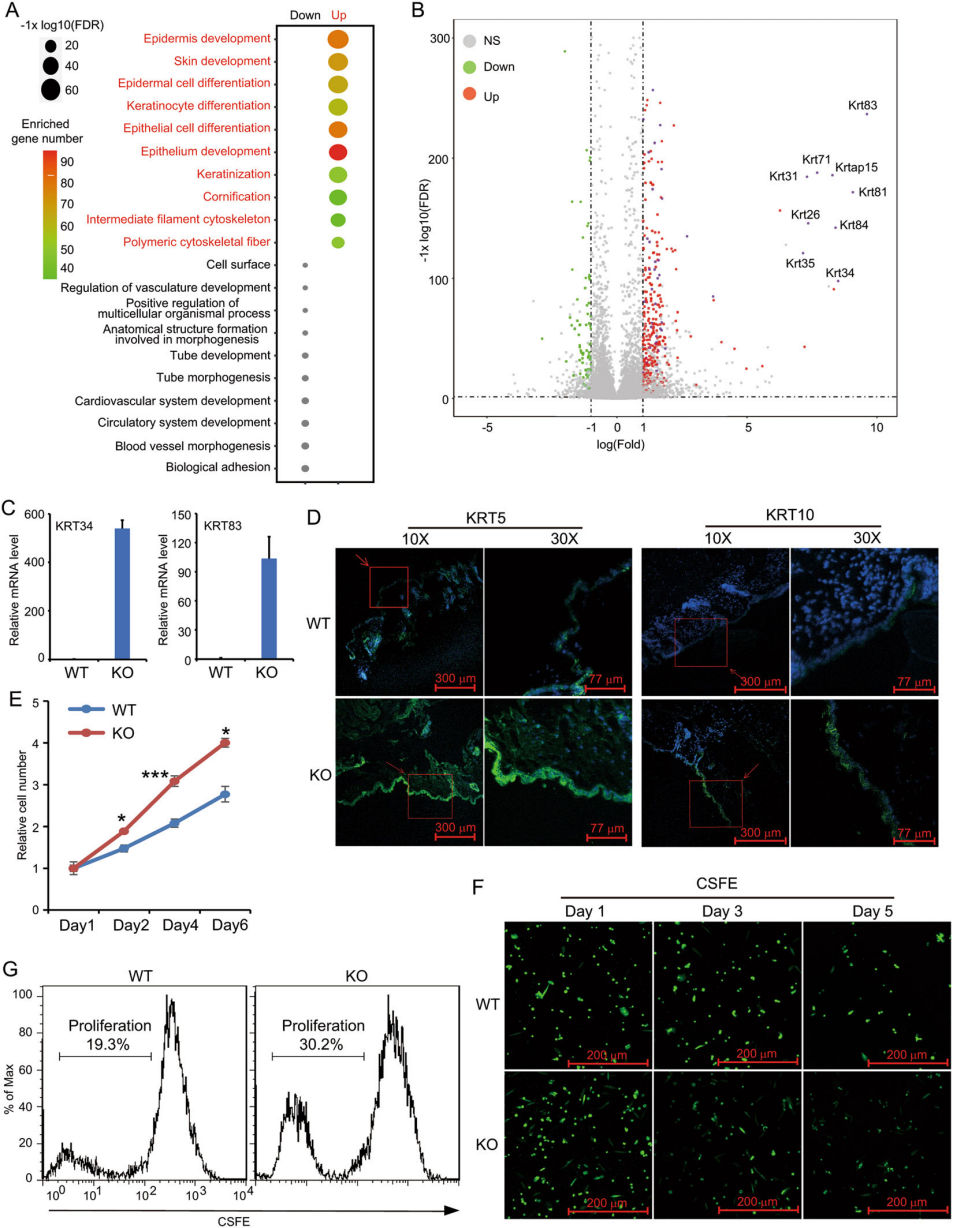
N4BP1 regulates keratinocytes proliferation and differentiation. **A** The most differentially expressed genes between N4BP1 wild-type and knockout skin were subjected to analyze the enrichment of GO biological process using online GSEA (geneset enrichment analysis) program. Both down- and upregulated genes from the enriched GO biological processes were shown. **B** Volcano plot was used to present the results of RNA-seq between N4BP1 wild-type and knockout back skin. The most-upregulated genes encoding keratins and keratin-associated proteins were shown. **C** The u-regulated genes encoding keratins including KRT34 and KRT83 were further examined in skin from N4BP1 wild-type and knockout mice by RT-PCR. **D** Immunofluorescence analysis of KRT5 and KRT10 in N4BP1 wild-type and knockout skin. **E** The cell number of keratinocytes isolated from N4BP1 wild-type and knockout was determined by CCK8 assay. **F** The isolated keratinocytes from N4BP1 wild-type and knockout mice skin were labeled with CFSE fluorescence and further cultured in indicated dates. The fluorescence was captured by fluorescence microscopy. **G** The CFSE labeled N4BP1 wild-type and knockout keratinocytes were cultured in vitro for 5 days. The fluorescence was examined by FACS. The images (**D, F, G**) show representative data from one of three independent experiments. Data (**C, E**) from one of three experiments are shown. Statistical differences between groups were determined by the Student’s *t*-test. *P < 0.05; **P < 0.01; ***P < 0.001.

Given that N4BP1 is an endoribonuclease that degrades mRNA, we carried out RNA sequencing in homeostatic WT and KO skin to further understand the molecular mechanism of N4BP1 in the regulation of keratinocytes. By profiling the mRNA between WT and KO mice, we identified 315 greatly upregulated and 78 greatly downregulated genes (FDR < 0.001, Expression > 10, and Fold change > 2) in KO mice skin. The upregulated but not downregulated genes are closely associated with epidermis development and keratinocyte differentiation (Fig. 3A). In the upregulated genes, there are a number of KRT genes such as proliferation marker (KRT5 and KRT14), differentiation marker (KRT1 and KRT10) as well as other KRT genes (KRT15, KRT26, KRT31, KRT34, KRT35, KRT72, KRT81, and KRT83) (Fig. 3B). The upregulation of KRT34, KRT71, and KRT83 was further confirmed by RT-PCR (Fig. 3C and Fig. S2A). We also did immunofluorescence to examine the protein expression of KRT5 and KRT10 in WT and KO skin (Fig. 3D). The results demonstrated a significant increase of both KRT5 and KRT10 in KO skin compared to them in WT skin (Fig. 3D). These results indicate that N4BP1-deficient keratinocytes have abnormal changes in molecular level, even though without obvious histological changes. Therefore, we reason that N4BP1 might be a critical regulator of keratinocyte proliferation and differentiation. To confirm this hypothesis, we isolated the primary keratinocytes from WT and KO skin. In in vitro culture, the proliferation rate of N4BP1-deficient keratinocytes was significantly higher than WT keratinocytes (Fig. 3E and Fig. S2B). Carboxyfluorescein succinimidyl ester (CFSE) labeling further verified that the cell division of N4BP1-deficient keratinocytes is much quicker than that of WT keratinocytes (Fig. 3F and G). Taken together, our results demonstrate that N4BP1 is a critical negative regulator in controlling keratinocyte proliferation.

### N4BP1 negatively regulates JunB and FosB

**Fig. 4:**
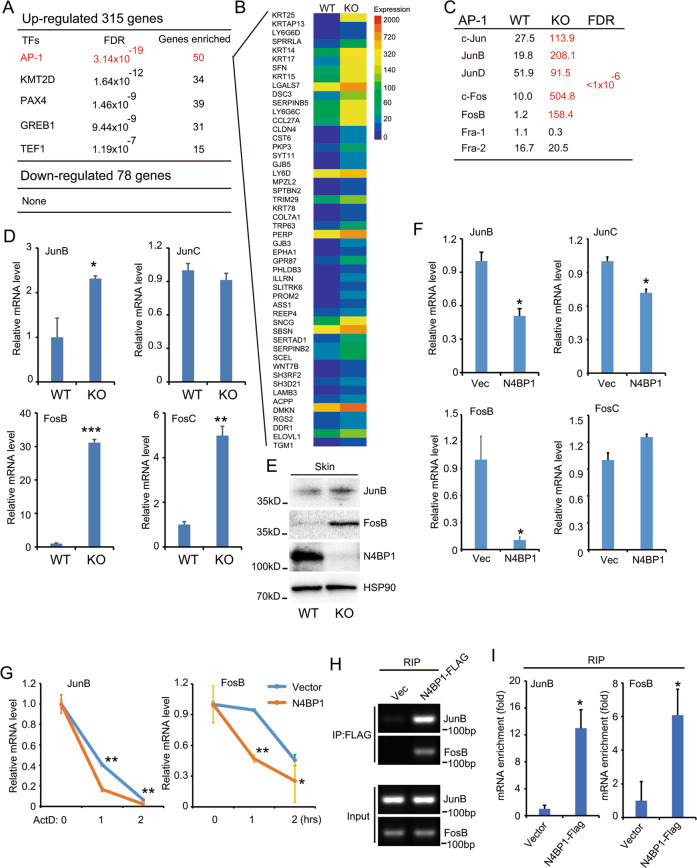
N4BP1 negatively regulates JUNB and FOSB. **A** The most up- and downregulated genes between N4BP1 wild-type and knockout mice skin were subjected to all transcription factor targets analysis (https://www.gsea-msigdb.org/). Among 315 most-upregulated genes, 50 genes are targets of AP-1 transcription factor. **B** The list of 50 targets of AP-1 and their expression change between N4BP1 wild-type and knockout mice skin. **C** The expression of AP-1 family (c-Jun, JunB, JunD, c-Fos, FosB, Fra-1, and Fra-2) in N4BP1 wild-type and knockout mice skin was examined by RNA-seq. **D** The mRNA level of JunB, JunC, FosB, and FosC was determined by real-time RT-PCR in N4BP1 wild-type and knockout mice skin. **E** The protein level of JunB and FosB was examined by Western analysis in N4BP1 wild-type and knockout mice skin. **F** The mRNA level of JunB, JunC, FosB, and FosC was determined by real-time RT-PCR in N4BP1 stable overexpressed HeLa cells. **G** The control and N4BP1 stable expressed HeLa cells were treated with actinomycin (20 uM) for indicated time and the mRNA of JunB and FosB were examined by real-time RT-PCR. **H** The RNA immunoprecipitation was performed using anti-FLAG antibodies in FLAG-N4BP1 stable overexpressed cells and their controls. The presence of RNA was determined by RT-PCR. **I** The RNA immunoprecipitation was performed by anti-FLAG antibodies in FLAG-N4BP1 stable overexpressed cells and their controls. The level of RNA was determined by real-time RT-PCR and normalized to the amount of input. The images (**E, H**) show representative data from one of three independent experiments. Data (**D, F, G, I**) from one of three experiments are shown. Statistical differences between groups were determined by the Student’s *t*-test. *P < 0.05; **P < 0.01; ***P < 0.001.

We next sought to determine the direct targets of N4BP1 in skin. Since 315 profoundly upregulated genes were identified in N4BP1-deficient skin. It is possible that these upregulated genes are controlled by key transcription factors (TFs), which are direct targets for N4BP1. Based on this consideration, we used transcriptional factor program (www.gsea-msigdb.org) to search the common TFs of 315 upregulated genes. Interestingly, there are 50 genes are transcriptional targets of AP-1 transcription factor (Fig. 4A). The enrichment is specific because the 78 downregulated genes are not enriched with any known TFs. The 50 targets of AP-1 were listed as in Fig. 4B and all of them are significantly upregulated in N4BP1-deficient skin. It is known that AP-1 is comprised of seven members including c-Jun, JunB, JunD, c-Fos, FosB, Fra-1, and Fra-2^34^. Based on the RNA-seq data, c-Jun, JunB, JunD, c-Fos, and FosB but not Fra-1 or Fra-2 are significantly upregulated in N4BP1-deficient skin (Fig. 4C). To further confirm these results, we examined the expression of c-Jun, JunB, c-Fos, and FosB in WT and KO skin by RT-PCR. As expected, the expression of JunB, FosB, and c-Fos is significantly elevated (Fig. 4D). Consistently, the protein levels of JunB and FosB are significantly upregulated in KO mice skin compared to that in WT mice skin (Fig. 4E). When overexpression of N4BP1, the expression of JunB, FosB, and JunC are significantly downregulated (Fig. 4F and Fig. S3A and S3B). These results indicate that JunB and FosB might be direct targets of N4BP1 and N4BP1 might control their mRNA stability. To further verify this hypothesis, we firstly examine the effect of N4BP1 on mRNA stability of JunB and FosB. As shown in Fig. 4G, the mRNA stability of JunB and FosB is greatly reduced in N4BP1 overexpressed cells. Then, we probe whether JunB and FosB is the direct targets of N4BP1 by RNA immunoprecipitation assay. Interestingly, both JunB and FosB mRNA were found in anti-FLAG-N4BP1 precipitation (Fig. 4H and I). It indicates that N4BP1 interacts with JunB and FosB mRNA. Furthermore, in 293 T cells, genetic knockout of N4BP1 results in upregulation of JunB and FosB as well as increased mRNA stability (Fig. S3C-S3E). Taken together, these results strongly suggest that JunB and FosB are direct targets of N4BP1 and N4BP1 controls their mRNA stability.

### Increased recruitment of neutrophil in psoriatic skin from N4BP1-deficient mice

**Fig. 5:**
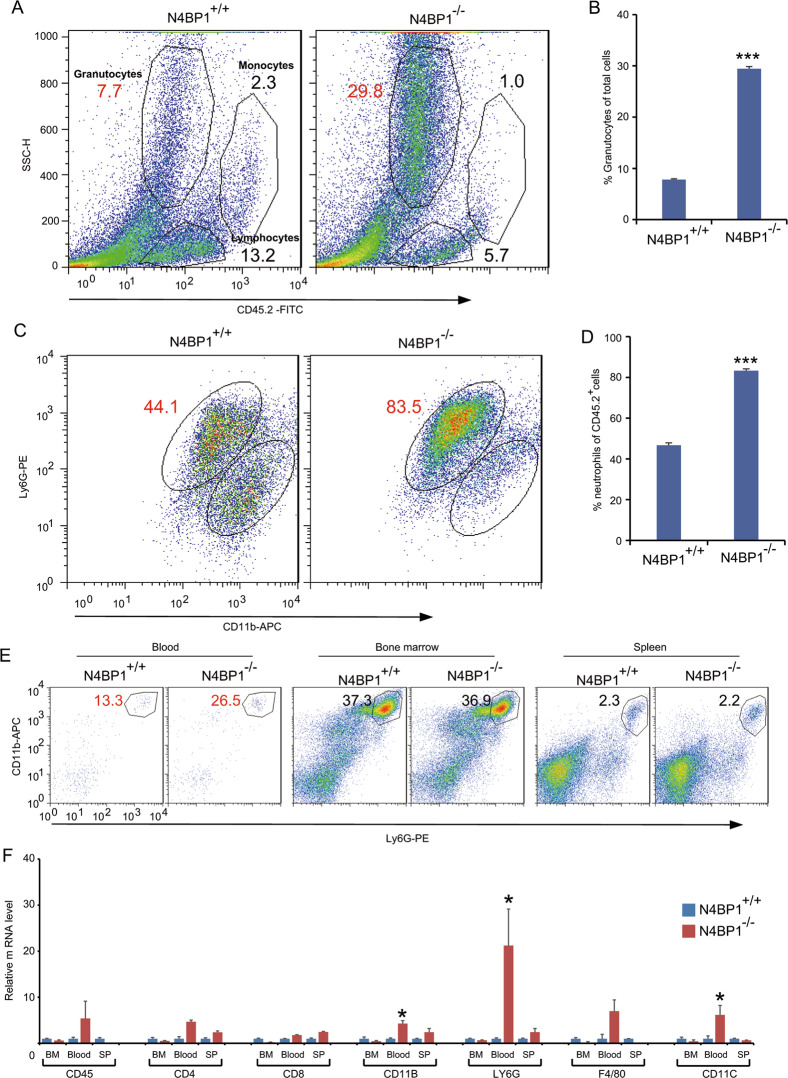
Increased neutrophil recruitment in N4BP1-deficient mice. **A** Cells were isolated from N4BP1 wild-type and knockout skin treated with IMQ. Cells were stained by CD45.2-FITC and analyzed by FACS. Shown are representative plots from one of three independent experiments. **B** The quantification of Granulocytes from **A**. **C** Cells were isolated from N4BP1 wild-type and knockout skin treated with IMQ. Cells were stained by CD45.2-FITC, LY6G-PE, and CD11B-APC antibodies, and analyzed by FACS. Shown are representative plots from one of three independent experiments. **D** The quantification of neutrophils from **C**. **E** Cells were isolated from N4BP1 wild-type and knockout mice blood and stained by CD45.2-FITC, LY6G-PE, and CD11B-APC antibodies then analyzed by FACS. **F** The total RNA from bone marrow, blood, and spleen of N4BP1 wild-type and knockout mice were extracted. The mRNA levels of genes including CD45, CD4, CD8, CD11B, LY6G, F4/80, and CD11C were determined by real-time RT-PCR. The images (**A, C, E**) show representative data from one of three independent experiments. Data (**F**) from one of three experiments are shown. Statistical differences between groups were determined by the Student’s *t*-test. *P < 0.05; **P < 0.01; ***P < 0.001.

As a chronic inflammatory disease, previous studies suggest that psoriasis is an abnormal process that results from extravagant crosstalk between keratinocytes and immune cells. Thus, we examined the effect of N4BP1 on immune function by checking the CD45-positive cell populations in psoriatic skin from WT and KO mice. The percentage of granulocytes in psoriatic skin from KO mice is much greater than that from WT control mice (Fig. 5A and B). Further analysis indicates that the increased granulocytes are CD11B and LY6G double-positive neutrophils (Fig. 5C and D). Next, we examined the percentage of neutrophils in bone marrow (BM), spleen, and blood from WT and KO mice. The results show that the percentage of neutrophil is dramatically increased in blood, but not in bone marrow and spleen, from KO mice compared to that from WT mice (Fig. 5E). To further prove this result, we analyzed the expression of molecular markers for different immune cell population in BM, blood, and spleen by RT-PCR. As expected, the mRNA level of LY6G is significantly higher in blood but not BM or spleen from KO mice compared to that of WT mice (Fig. 5F). These data indicate that N4BP1 deficiency results in increased recruitment of neutrophil in psoriatic skin and elevated percentage of neutrophil in blood.

### The number and maturation of neutrophils is significantly increased in N4BP1-deficient mice

**Fig. 6:**
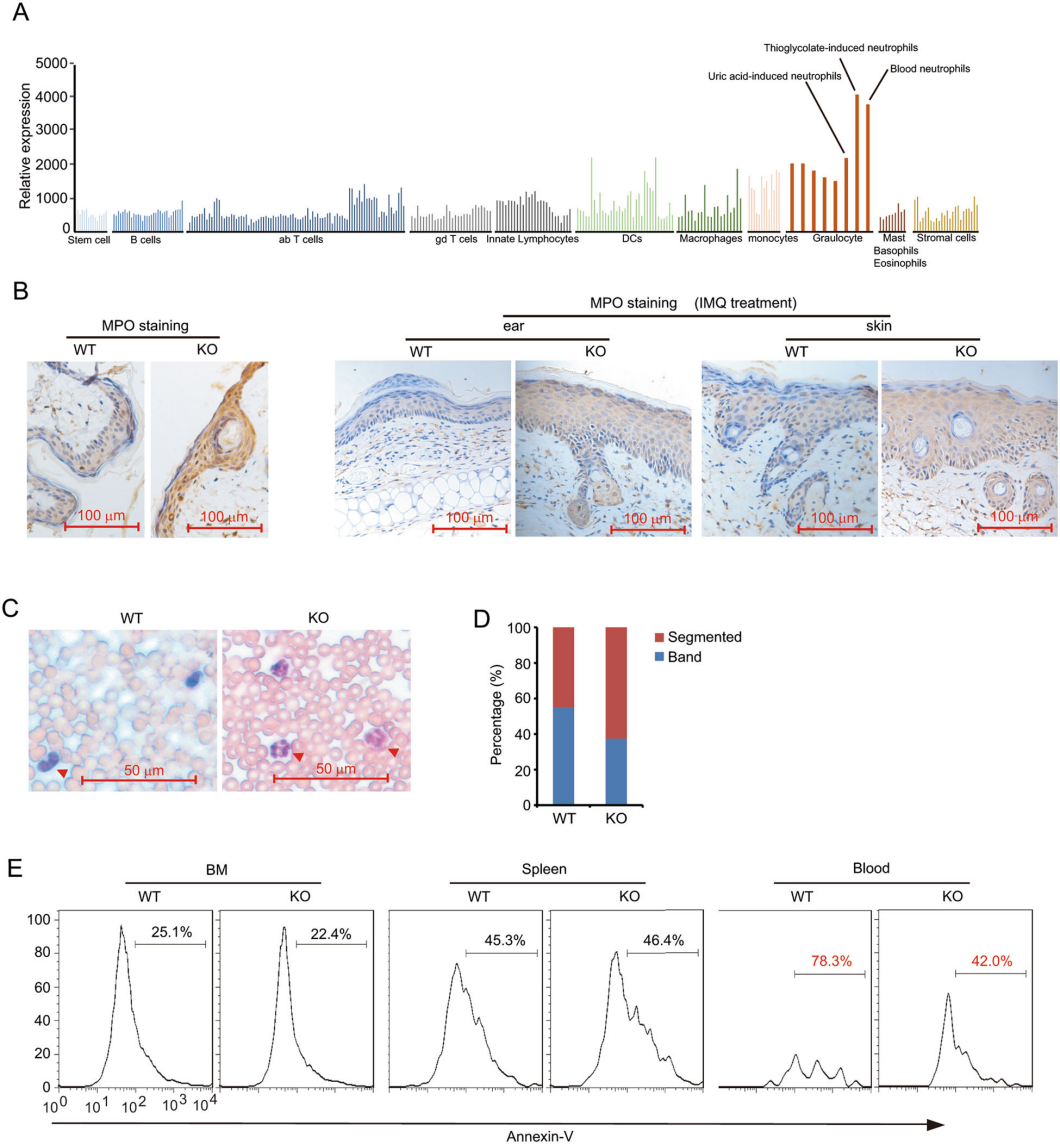
N4BP1 controls neutrophil maturation. **A** The expression level of immune cells was loaded from the immunological genome project (www.immgen.org). The figure was regenerated and reorganized. N4BP1 shows highest expression in neutrophil among all immune cell subtypes. **B** MPO staining in N4BP1 wild-type and knockout mice skin with or without IMQ treatment. **C** Giemsa stain is used to examine the neutrophil of the blood smear from N4BP1 wild-type and knockout mice blood. **D** The percentage of segmented and band neutrophil in the blood smear from N4BP1 wild-type and knockout mice blood. **E** Cells from bone marrow, spleen, and blood were isolated and cultured in vitro for 24 h. Then these cells were stained by CD45.2, LY6G, and Annexin-V antibodies. The percentage of Annexin-V-positive neutrophils were analyzed by FACS. The images (**B, C, E**) show representative data from one of three independent experiments.

The enhanced neutrophil in blood and psoriatic skin from KO mice prompted us to further detect the function of N4BP1 in neutrophils. By mining the expression of N4BP1 in all mouse immune cell types, we found that neutrophils, especial blood neutrophils has the highest expression of N4BP1 in all tested immune cells (Fig. 6A, www.immgen.org). Myeloperoxidase (MPO) is a key constituent of the neutrophil’s cytotoxic armament. Thus, we performed MPO staining both in resting and IMQ-induced skin and found that the level of MPO in KO skin is significantly increased (Fig. 6B). We then did the blood smear to check the abnormalities in the number and shape of neutrophil from WT and KO mice. Both the neutrophil number and the percentage of segmented neutrophil in KO mice are greatly elevated (Fig. 6C and D). Interestingly, we further found that the apoptotic neutrophil from KO mice blood, but not bone morrow or spleen, is significantly decreased compared to it from WT mice (Fig. 6E). These results suggest that the increased infiltration of neutrophil in KO skin at least partially due to increased segmented neutrophil in blood.

### N4BP1 negatively controls the mRNAs of CXCL1, CCL20, S100A8, and S100A9

**Fig. 7:**
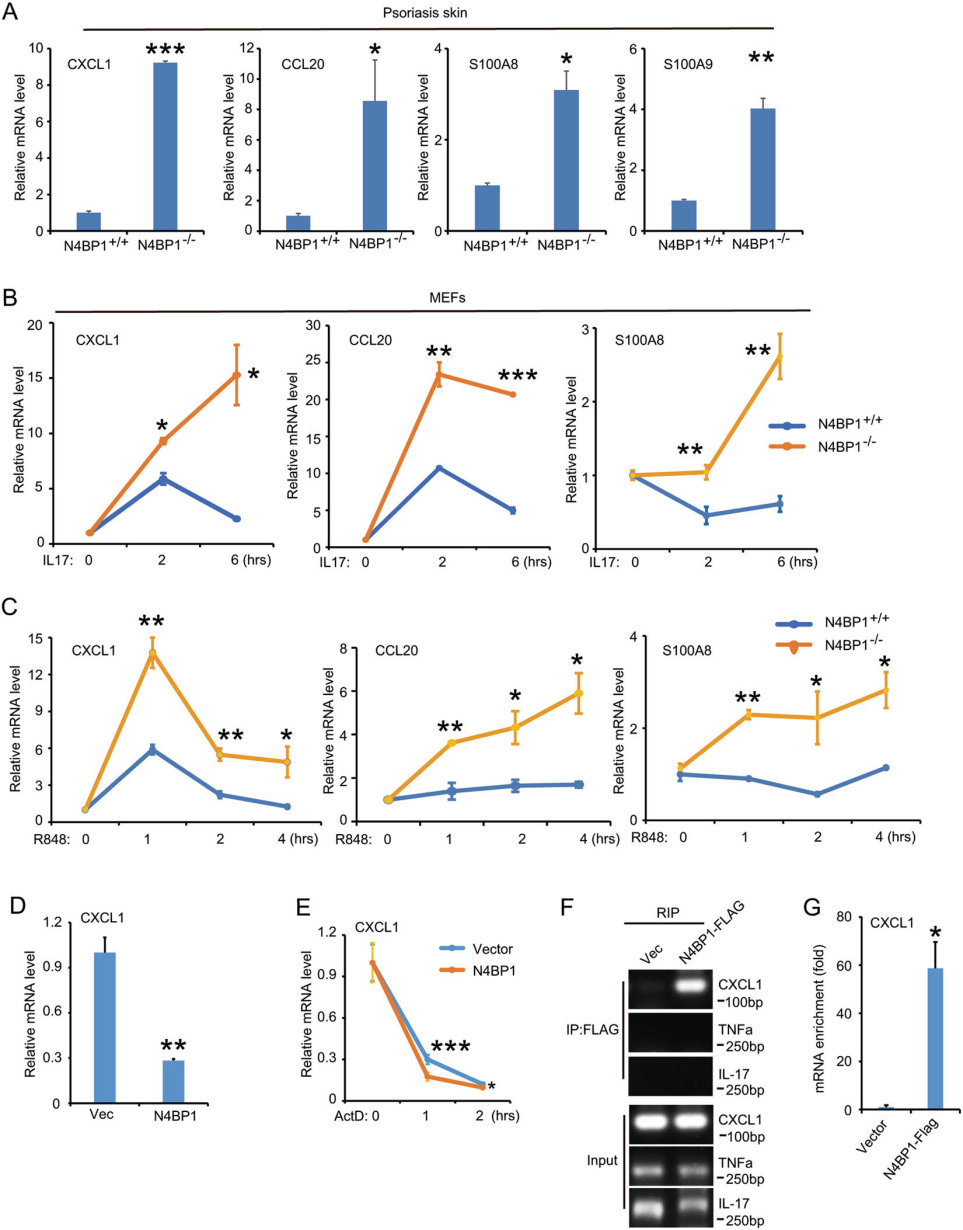
N4BP1 negatively regulates CXCL1 CCL20, S100A8, and S100A9. **A** The mRNA level of CXCL1, CCL20, S100A8, and S100A9 was determined in psoriatic skin from N4BP1 wild-type and knockout mice. **B** MEFs were isolated from N4BP1 wild-type and knockout mice and treated with IL-17 (20 ug/ml) for indicated hours. The mRNA level of CXCL1, CCL20, and S100A8 was determined by real-time RT-PCR. **C** The N4BP1 wild-type and knockout MEFs were treated with R848 (10 uM) for indicated hours. The mRNA level of CXCL1, CCL20, and S100A8 was determined by real-time RT-PCR. **D** The mRNA level of CXCL1 was determined by real-time RT-PCR in N4BP1 stable overexpressed HeLa cells. **E** The control and N4BP1 stable expressed HeLa cells were treated with actinomycin (20 uM) for indicated time and the mRNA of CXCL1 were examined by real-time RT-PCR. **F** The RNA immunoprecipitation was performed using anti-FLAG antibodies in FLAG-N4BP1 stable overexpressed cells and their control cells. The presence of RNA (CXCL1, TNFa, and IL-17) was determined by RT-PCR. **G** The RNA immunoprecipitation was performed using anti-FLAG antibodies in FLAG-N4BP1 stable overexpressed cells and their control cells. The level of RNA was determined by real-time RT-PCR and normalized to the amount of input. The images (**F**) show representative data from one of three independent experiments. Data (**A, B, C, D, E, G**) from one of three experiments are shown. Statistical differences between groups were determined by the Student’s *t*-test. *P < 0.05; **P < 0.01; ***P < 0.001.

To understand the molecular mechanism of N4BP1 in the regulation of neutrophil infiltration, we examined the mRNA level of several key genes involved in inflammation including TNFa, IL-23, IL-17, CXCL1, CCL20, S100A8, and S100A9 in psoriasis skin from N4BP1 KO and WT mice. Consistent with increased neutrophils, the expression of CXCL1, CCL20, S100A8, and S100A9 but not TNFa, IL-17, and IL-23 are significantly increased in the psoriasis skin from KO mice (Fig. 7A and data not shown). CXCL1 and CCL20 are IL-17-induced genes. Treatment with IL-17 in MEFs significantly induces higher expression of CXCL1, CCL20, and S100A8 in N4BP1 KO MEFs comparing to that in WT MEFs (Fig. 7B). Using another TLR8 agonist, R848, we got similar results (Fig. 7C). These results indicate that CXCL1, CCL20, S100A8, and S100A9 might be direct targets of N4BP1 to control neutrophil maturation and infiltration. Overexpression of N4BP1 significantly downregulates the mRNA level of CXCL1 (Fig. 7D). When treated cells with ActD at indicated timepoints, the relative mRNA of CXCL1 is significantly lower in N4BP1 overexpressed cells comparing to that in control cells (Fig. 7E). Moreover, RNA immunoprecipitation showed that FLAG-N4BP1 binds with CXCL1 (Fig. 7F and G).Upon R848 treatment, the KO keratinocytes proliferate much faster than WT controls as examined by CFSE labeling (Fig. S4). Taken together, loss of N4BP1 results in not only abnormal keratinocyte proliferation but also abnormal neutrophil maturation and infiltration by directly controlling JunB, FosB, and CXCL1, respectively, leading to susceptibility to psoriasis.

## Discussion

Psoriasis is a serious and chronic skin disease with no clear cause and cure that affects more than 100 million individuals in the world (www.WHO.int). The development of psoriasis is a complicated process resulting from abnormal interplay between keratinocytes and immune cells. Although the role of T cells in psoriasis has been extensively studied and significant progress both in pathology and treatment has been achieved in recent years^35,36^, The intrinsic roles of keratinocytes as well as immune cells other than T cells remains to be fully explored in psoriasis. Here, we recognized N4BP1 is a critical endoribonuclease in both keratinocytes and neutrophils. With predominate expression in keratinocytes and neutrophils, N4BP1 regulates keratinocytes and neutrophils through controlling JunB, FosB, and CXCL1, respectively. Although we did not observe gross abnormality of skin in N4BP1-deficient mice, changes in both cellular and molecular levels of keratinocytes and neutrophils have taken place in the N4BP1-deficient skin (Figs. 3, 4, and 5). Hence, N4BP1-deficient mice skin is already in a state to favor keratinization and cornification. These molecular and cellular changes in keratinocytes and neutrophils are more apt to development of psoriasis upon IMQ stimulation. Therefore, our study provides compelling evidence that N4BP1 plays a key role in skin hemostasis and pathology through regulating keratinocytes and neutrophils. N4BP1 acts as a nuclear ribonuclease to take charge of keratinocytes and neutrophils, and its abnormality contributes to psoriasis development, presenting it as a potential therapeutic target to treat psoriasis.

TNFa, IL-17, and IL-23 are three well-recognized cytokines that play important role in psoriasis. These three cytokines are efficient targets in the treatment of psoriasis in clinic. For example, Brodalumab is the anti-IL17RA antibody and has been approved for use in patients with moderate to severe plaque psoriasis^37^. About 40% of patients receiving 12 week of induction therapy with subcutaneous brodalumab achieve a complete skin clearance (PASI 100). Certolizumab is the anti‐TNFa biologic and about 50% of patients reach PASI-90 after a long-term treatment^38^. Risankizumab is a humanized monoclonal antibody that specifically targets interleukin (IL)-23 and around 50% patients treated with subcutaneous risankizumab show a clear skin^39^. Thus, although some patients have benefited, a significant portion of patients still cannot reach a completely clear skin even after extensive treatment with newest biologics. It indicates that there are still other unknown factors significantly contribute to the development and recurrence besides these critical inflammatory cytokines (TNFa, IL-23, and IL-17). In N4BP1-deficient mice under resting- or IMQ-treated condition, the expression of TNFa, IL-23, and IL-17 is similar or slightly downregulated comparing to their WT controls (data not shown). It suggests that the severe psoriasis observed in N4BP1-deficient mice is not due to overproduction of TNFa, IL-23, and IL-17. However, in the IL-17-treated cells, the target genes such as CXCL1, CCL20, and S100A8 in N4BP1-deficient cells are higher than it in controls (Fig. 7B). It indicates that N4BP1 regulates cytokine signal transduction instead of the production of cytokines. It suggests that N4BP1 is a novel regulator in psoriasis and its functional is independent on overproduction of TNFa, IL-23, and IL-17.

N4BP1 has a NYN domain and the primary amino-acid sequence as well as structure is similar to that of Regnase-1^33^. Regnase-1 is not only a RNA binding protein but also an endoribonuclease that is essential for degradation of inflammation-related mRNAs such as IL-6 and maintenance of immune homeostasis^26,40–42^. Regnase-1 specifically cleaves and degrades translationally active mRNAs via recognition of the stem loop structure in 3’UTR at ribosome/endoplasmic reticulum^43^. Our results reported here also suggests that N4BP1 degrades specific mRNAs such as JunB, FosB, and CXCL1. But, it is still unclear how N4BP1 achieve substrate specificity and whether N4BP1 also recognizes stem loop structure in 3’UTRs. Regnase-1 is an IL-17-induced gene and its upregulation in psoriasis restricts IL-17 signaling and skin inflammation^29,30^. Keratinocyte-specific ablation of Regnase-1 promotes local and systemic inflammation partially through inhibiting the IL-36 function^44,45^. But unlike Regnase-1, N4BP1 mainly localizes on nucleus and thus might degrade different mRNAs and controls different biological processes. Our results indicate that N4BP1 plays critical and nonredundant role in psoriasis via controlling the function of keratinocytes and neutrophils.

Munro microabscesses (intracorneal aggregates of neutrophils) in chronic plaque psoriasis and spongiform pustules of Kogoj (epidermal spongiosis with neutrophils) in pustular psoriasis are histologic hallmarks of psoriasis^46–48^. Neutrophil was first observed from psoriatic lesions in 1898, but its role in psoriasis is still puzzled^21^. In N4BP1-deficient skin after IMQ induction, the dominant infiltrated immune cells are neutrophils (Fig. 5A). This is partially due to overproduction of neutrophil-chemokine CXCL1 in IL-17-induced keratinocytes. Interestingly, N4BP1 also involved in the circulation of neutrophil in the blood. We observed increased total number of neutrophil in blood and the percentage of segmented neutrophil is further increased (Fig. 6C). Although, the direct molecular targets of N4BP1 in controlling neutrophil maturation and survival are still unknown. We do observed a reduction of apoptotic neutrophil in N4BP1-deficient mice. Therefore, N4BP1 is a key regulator in the maturation and survive of neutrophils. Loss of N4BP1 results in increased segmented neutrophil in blood and contributes to the development of psoriasis.

During the process of manuscript submission, Gitlin et al. reported that N4BP1 is a negative regulator of cytokine production and its function can be disrupted by FADD/caspase-8-mediated cleavage^49^. They established the N4BP1 knockout mice and examined the response of these mice under IMQ-induced psoriasis model. They observed that the N4BP1-deficient mice developed exacerbated psoriasis with more epidermal hyperplasia, inflammatory cell infiltration, and serum CXCL1 level compared to WT mice^49^. Here, we independently established the N4BP1 knockout mice and performed detailed analysis to uncover the role of N4BP1 in psoriasis. In IMQ-induced psoriasis model, we found N4BP1 knockout mice developed severe psoriasis due to abnormal keratinocyte proliferation and neutrophil infiltration. N4BP1 binds to its mRNA targets including JunB, FosB, and CXCL1, and controlling their stability. The back-to-back investigations performed by two groups support the important role of N4BP1 in psoriasis.

In summary, we uncovered a new molecule involved in psoriasis. In fact, as a member of Zc3h12a-like ribonuclease-NYN domain subfamily of endoribonucleases, Regnase-1 has been reported to play a role in the keratinocytes. Here, we unraveled another member that is N4BP1, plays critical role both in keratinocytes and neutrophils by directly controlling mRNA stability of several key genes including JunB, FosB, and CXCL1, and through such mechanism maintains skin hemostasis to prevent the development of psoriasis (Fig. S5). It is possible that N4BP1 might be cooperatively with Regnase-1 to regulate distinguished mRNA targets. Further studies on the detailed regulatory mechanism of how N4BP1 specifically recognizes its targets and its relationship with Regnase-1 should provide additional insights into the pathogenesis of psoriasis.

## Materials and methods

### Cell lines and generation of N4BP1 knockout mice

HEK293T cells and HeLa cells were cultured with modified DMEM high glucose medium (containing 10% FBS and 1% streptomyces and penicillin) under 37 °C and 5% of CO2. Lipofectamine 2000 was used for transfection and the medium was replaced after 6 h of transfection. Forty-eight hours after transfection, puromycin was used to screen successfully transfected cells and maintained for 2 weeks. WT and N4BP1^−/−^ MEFs were prepared from day 13.5 embryos and cultured in DMEM supplemented with 10% FBS.

To obtain the N4BP1 knockout mice, we designed two sgRNAs to specifically delete the second exon of N4BP1 encoding gene. The procedure to generate N4BP1 knockout mice was performed by Shanghai Model Organisms Center Inc. N4BP1^+/−^ mice were intercrossed to generate N4BP1^−/−^ mice and backcrossed more than 10 generations onto the C57BL/6 genetic background. The sample size of mouse for different experiments was designed based on the results of primary experiments and the cost, time, or convenience of collecting the data, as well as the need for it to offer sufficient statistical power. The mice were housed at the institute of experimental animal sciences at Nantong University. All studies and procedures were approved by the animal research committee of Nantong University. The mouse genotype was determined by using the genomic DNA of tails. The genomic PCR was performed using kit of Taq Master Mix (Vazyme,#p112-01) and the PCR products were separated by the 1% of agarose gel. The sequence of primers is as follows: F1: GGAGATGTACGGC CACCAGAG, R1: CCTCTT GTGACAAACTG, F2: TGCTAAGCCATCTCACCAGA, R2: GTGAA GGGGAGGAGAGGAAG.

### Primary keratinocyte isolation and culture in vitro

The neonatal mice were sacrificed and skins from the limbs were separated. The separated skins were washed with 75% of alcohol for 2 min, then rinsed in PBS with antibiotics for two times. The skins were flattened in 3-cm petri dishes and digested with dispase (StemCell Technologies, 07913) for 18 h at 4 °C. The epidermis was taken off gently and were cut into small pieces. The epidermis was further digested with 0.25% trypsin containing EDTA at 37 °C for 15 min. The digested epidermis was shaken gently and the keratinocytes were collected by centrifugation. The isolated keratinocytes were washed with keratinocyte culture medium and cultured with complete medium of mouse epidermal keratinocytes (Procell, CM-M094). Keratinocytes were cultivated at 37 °C with 5% CO_2_, and the medium was refreshed every 2 days. The proliferation of primary keratinocytes was determined by CCK8 assay. Keratinocytes were labeled with CFSE (carboxyfluorescein succinimidyl ester) using the Cell Trace Cell proliferation kit (Thermo Fisher, C34570). Briefly, the primary keratinocytes were gently suspended with CFSE (1:1000 diluted). After incubating at 37 °C for 20 min, the complete medium was added and incubated for additional 5 min. The supernatant was removed and the cells were cultured in complete medium for indicated days. The labeled cells were assayed using cytometry and fluorescent microscopy.

### Cutaneous cell preparation and analyzed by flow cytometry

The back skins were collected from N4BP1^+/+^ and N4BP1^−/−^ mice with or without IMQ (Aldara cream, 5%) treatment. The skins were cut into small pieces and further mechanically homogenized. To obtain single-cell suspension, these skins were digested at trypsin at 37 °C for 90 min. The cell suspension were filtered through the 100 uM filter and collected by centrifugation. Cells were stained with antibodies according to the manufacturer’s instructions. Cells were detected by BD FACSC Calibur flow cytometry and analyzed using FlowJo 7.6 software.

### Establishment of psoriatic mouse model

The back hair of N4BP1^+/+^ and N4BP1^−/−^ mice were removed using an electric razor. The 8–10-weeks mice were received a daily topical dose of 62.5 mg of 5% IMQ cream (corresponding to 3.125 mg of active compound) (Aldara cream, 5%) for up to 11 consecutive days. At designed time, the IMQ-treated N4BP1^+/+^ and N4BP1^−/−^ mice were sacrificed and the skins were isolated for H&E or immunohistochemical staining.

### Hematoxylin-eosin and immunohistochemical staining

The skin tissue was trimmed into small pieces at size about 1 × 1 cm. Then the skin tissues were soaked in 4% paraformaldehyde for 24 h following alcohol gradient dehydration and paraffin embedding. The tissue blocks were cut into 5-um-thick slices. Sections were dewaxed, hydrated and stained with hematoxylin for 30 min and eosin for 5 s. At last, the stained sections were dehydrated, transparent and sealed. For immunohistochemical staining, the thick slices of 5 um were dewaxed with xylene and soaked in water. In order to reduce the nonspecific staining caused by endogenous peroxidase, 3% hydrogen peroxide was dripped on the slices and placed at room temperature for 30 min. Then, goat serum was used to block nonspecific binding for 1 h at room temperature. The sections were rinsed with TBST buffer and incubated with primary antibody at 4 °C overnight. Next day, the anti-mouse or anti-rabbit IgG conjugated with horseradish peroxidase was incubated at 37 °C for 30 min. Sections stained with **DAB** (3,3’-diaminobenzidine) for 2 min and the nucleus was stained with hematoxylin for 5 min.

### Blood smear and Giemsa staining

A drop of the fresh peripheral blood or bone marrow from N4BP1^+/+^ and N4BP1^−/−^ mice were placed on the clean slide. Without delay, place a spreader at an angle of 45° from the slide and move it back to make contact with the drop. The slides were dried and then fixed with methanol for 15 min. Then the blood smear was stained with Giema dye for 30 min. The stained slides were washed with buffer and dried. The stained blood smear was observed under the microscope.

### Immunofluorescence staining

The tested cells were inoculated into 2 cm petri dishes with round slides placed in advance. When the cell density reached 60% on the round glass slides, slides were taken out and fixed with 4% paraformaldehyde (PFA) for 15 min, and permeabilized by 0.2% Triton X-100. The fixed cells were blocked with 1% BSA and washed with PBS plus 0.1% BSA and 0.01% Tween-25. After that, cells were stained with anti-N4BP1 antibody overnight at 4 °C. At next day, the slides were washed and further stained with second antibody at room temperature for 2 h. After PBS washing, the Hochest 33343 was used to for nuclear staining. The fluorescence of stained cells were analyzed by confocal microscope or fluorescence microscope.

### RNA isolation, quantitative real-time PCR, and RNA-sequence

Skin samples or other tissue samples were collected and placed in 1.5-ml tube for total RNA extraction. For RNA isolation, the sample is cracked in the lysate buffer by a homogenizer. The purity and concentration of total RNA were assessed using a Nano Drop 1000 spectrophotometer (Thermo Fisher Scientific). Reverse transcription was performed with 1 μg of total RNA using HiScript II One Step RT-PCR Kit. The cDNA was diluted five times, and SYBR Green PCR Kit (#Q112-02/03, Vazyme) was used to quantitative the expression of gene. The mRNA level was normalized against 18 S. The sequences of the primers are listed in Supplementary Table S1. For RNA sequencing, total RNAs extracted from tissues and send to company for RNA-seq. The analysis about enrichment and targets of transcription factor was performed using online GSEA program (www.gsea-msigdb.org).

### RNA immunoprecipitation

The cells were collected, washed twice with precooled PBS to remove the medium, and then 10 ml of PBS was added. Add formaldehyde (37% stock solution) to the above solution to a final solution concentration of 1% and incubate with slow rotation at room temperature for 10 min. Add glycine solution to a final concentration of 0.25 M to quench the crosslinking reaction (pH 7.0), then incubate at room temperature for 5 min and mix gently. Add 5–10 ml of precooled PBS to wash the cells and scrape the cells in 2 ml of precooled PBS. The cells were collected by centrifugation and washed twice with cold PBS. The collected cells were lysated by RIPA lysis buffer with PMSF and sonicated. After rotation for 1 h, the lysate was centrifuged at 4°C, 13,000 rpm. The supernatant was taken and precleaned by protein A beads. After centrifugation, the clean supernantant were incubated with anti-FLAG antibodies for 2 h. Then, protein A was added for additional 1 h. After that, protein A beads were collected and washed. The immunoprecipitated samples were resuspended in decrosslinking agent at 70 °C for 45 min to reverse crosslinking. After reverse crosslinking, Trizol was used for RNA extract.

### Western blot analysis

Briefly, ultrasonic lysis of skin tissue in RIPA lysate with PMSF, samples were further homogenized in a lysis buffer and centrifuged at 10,000 *g*, 4 °C for 15 min to collect the supernatant. Protein concentrations were determined by a BCA protein assay kit (Bio-Rad). Subsequently, the supernatant was diluted in 5× SDS loading buffer and boiled for 15 min. The protein samples were separated with 10% SDS-polyacrylamide gel electrophoresis (SDS-PAGE) and transferred to polyvinylidene difluoride filter (PVDF) membranes (Millipore), which were blocked in 5% milk dissolved in Tris buffered saline containing 0.05% Tween 20. Thereafter, the membranes were incubated with primary antibodies at first and then the secondary antibody linked with horseradish peroxidase for 2 h. The detection of chemiluminescent signals was performed by ECL method (Bio-Rad). The Antibodies are listed in Supplementary Table S2.

## Supplementary information

Supplemental Figure Legends

Supplemental Figure S1

Supplemental Figure S2

Supplemental Figure S3

Supplemental Figure S4

Supplemental Figure S5

## References

[1] Nestle FO, Kaplan DH, Barker J (2009). Psoriasis. N. Engl. J. Med..

[2] Rendon A, Schakel K (2019). Psoriasis pathogenesis and treatment. Int. J. Mol. Sci..

[3] Billi AC, Gudjonsson JE, Voorhees JJ (2019). Psoriasis: past, present, and future. J. Invest. Dermatol..

[4] Boehncke WH, Brembilla NC (2018). Unmet needs in the field of psoriasis: pathogenesis and treatment. Clin. Rev. Allergy Immunol..

[5] Furue M, Furue K, Tsuji G, Nakahara T (2020). Interleukin-17A and keratinocytes in psoriasis. Int. J. Mol. Sci..

[6] Kim HJ, Lebwohl MG (2019). Biologics and psoriasis: the beat goes on. Dermatol. Clin..

[7] Schon MP, Erpenbeck L (2018). The interleukin-23/interleukin-17 axis links adaptive and innate immunity in psoriasis. Front. Immunol..

[8] Reynolds KA, Pithadia DJ, Lee EB, Liao W, Wu JJ (2020). Safety and effectiveness of anti-tumor necrosis factor-alpha biosimilar agents in the treatment of psoriasis. Am. J. Clin. Dermatol..

[9] Ni X, Lai Y (2020). Keratinocyte: a trigger or an executor of psoriasis?. J. Leukoc. Biol..

[10] Benhadou F, Mintoff D, Del Marmol V (2019). Psoriasis: keratinocytes or immune cells—which is the trigger?. Dermatology.

[11] Casciano F, Pigatto PD, Secchiero P, Gambari R, Reali ET (2018). Cell hierarchy in the pathogenesis of psoriasis and associated cardiovascular comorbidities. Front. Immunol..

[12] Ippagunta SK (2016). Keratinocytes contribute intrinsically to psoriasis upon loss of Tnip1 function. Proc. Natl Acad. Sci. USA.

[13] Zenz R (2005). Psoriasis-like skin disease and arthritis caused by inducible epidermal deletion of Jun proteins. Nature.

[14] Singh K (2018). JunB defines functional and structural integrity of the epidermo-pilosebaceous unit in the skin. Nat. Commun..

[15] Pasparakis M (2002). TNF-mediated inflammatory skin disease in mice with epidermis-specific deletion of IKK2. Nature.

[16] Dancey JT, Deubelbeiss KA, Harker LA, Finch CA (1976). Neutrophil kinetics in man. J. Clin. Invest..

[17] Chiang CC, Cheng WJ, Korinek M, Lin CY, Hwang TL (2019). Neutrophils in psoriasis. Front. Immunol..

[18] Herster F (2020). Neutrophil extracellular trap-associated RNA and LL37 enable self-amplifying inflammation in psoriasis. Nat. Commun..

[19] Paliogiannis P (2019). Associations between the neutrophil-to-lymphocyte and the platelet-to-lymphocyte ratios and the presence and severity of psoriasis: a systematic review and meta-analysis. Clin. Exp. Med..

[20] Han G, Havnaer A, Lee HH, Carmichael DJ, Martinez LR (2020). Biological depletion of neutrophils attenuates pro-inflammatory markers and the development of the psoriatic phenotype in a murine model of psoriasis. Clin. Immunol..

[21] Mrowietz U (2017). Neutrophils’ sexiness is independent of trendy fashion. Exp. Dermatol..

[22] Tomecki R, Dziembowski A (2010). Novel endoribonucleases as central players in various pathways of eukaryotic RNA metabolism. RNA.

[23] Labno A, Tomecki R, Dziembowski A (2016). Cytoplasmic RNA decay pathways—enzymes and mechanisms. Biochim. Biophys. Acta.

[24] Anantharaman V, Aravind L (2006). The NYN domains: novel predicted RNAses with a PIN domain-like fold. RNA Biol..

[25] Habacher C, Ciosk R (2017). ZC3H12A/MCPIP1/Regnase-1-related endonucleases: an evolutionary perspective on molecular mechanisms and biological functions.. Bioessays.

[26] Matsushita K (2009). Zc3h12a is an RNase essential for controlling immune responses by regulating mRNA decay. Nature.

[27] Kidoya H (2019). Regnase-1-mediated post-transcriptional regulation is essential for hematopoietic stem and progenitor cell homeostasis. Nat. Commun..

[28] Yu F (2013). Bone marrow deficiency of MCPIP1 results in severe multi-organ inflammation but diminishes atherogenesis in hyperlipidemic mice. PLoS ONE.

[29] Monin L (2017). MCPIP1/Regnase-1 restricts IL-17A- and IL-17C-dependent skin inflammation. J. Immunol..

[30] Ruiz-Romeu E (2016). MCPIP1 RNase is aberrantly distributed in psoriatic epidermis and rapidly induced by IL-17A. J. Invest. Dermatol..

[31] Murillas R, Simms KS, Hatakeyama S, Weissman AM, Kuehn MR (2002). Identification of developmentally expressed proteins that functionally interact with Nedd4 ubiquitin ligase. J. Biol. Chem..

[32] Oberst A (2007). The Nedd4-binding partner 1 (N4BP1) protein is an inhibitor of the E3 ligase Itch. Proc. Natl Acad. Sci. USA.

[33] Yamasoba D (2019). N4BP1 restricts HIV-1 and its inactivation by MALT1 promotes viral reactivation. Nat. Microbiol.

[34] Karin M, Liu Z, Zandi E (1997). AP-1 function and regulation. Curr. Opin. Cell Biol..

[35] Karczewski J, Dobrowolska A, Rychlewska-Hanczewska A, Adamski Z (2016). New insights into the role of T cells in pathogenesis of psoriasis and psoriatic arthritis. Autoimmunity.

[36] Diani M, Altomare G, Reali E (2016). T helper cell subsets in clinical manifestations of psoriasis. J. Immunol. Res..

[37] Blair HA (2018). Brodalumab: A review in moderate to severe plaque psoriasis. Drugs.

[38] Gordon KB (2020). Long-term efficacy of certolizumab pegol for the treatment of plaque psoriasis: 3-year results from two randomized phase III trials (CIMPASI-1 and CIMPASI-2). Br. J. Dermatol.

[39] Blair HA (2020). Risankizumab: A review in moderate to severe plaque psoriasis. Drugs.

[40] Mao R (2017). Regnase-1, a rapid response ribonuclease regulating inflammation and stress responses. Cell Mol. Immunol..

[41] Iwasaki H (2011). The IkappaB kinase complex regulates the stability of cytokine-encoding mRNA induced by TLR-IL-1R by controlling degradation of regnase-1. Nat. Immunol..

[42] Jeltsch KM (2014). Cleavage of roquin and regnase-1 by the paracaspase MALT1 releases their cooperatively repressed targets to promote T(H)17 differentiation. Nat. Immunol..

[43] Mino T (2015). Regnase-1 and Roquin regulate a common element in inflammatory mRNAs by spatiotemporally distinct mechanisms. Cell.

[44] Takaishi M, Satoh T, Akira S, Sano S (2018). Regnase-1, an immunomodulator, limits the IL-36/IL-36R autostimulatory loop in keratinocytes to suppress skin inflammation. J. Invest. Dermatol.

[45] Konieczny P (2019). Keratinocyte-specific ablation of Mcpip1 impairs skin integrity and promotes local and systemic inflammation. J. Mol. Med. (Berl.).

[46] Liang Y, Sarkar MK, Tsoi LC, Gudjonsson JE (2017). Psoriasis: a mixed autoimmune and autoinflammatory disease. Curr. Opin. Immunol..

[47] Naik HB, Cowen EW (2013). Autoinflammatory pustular neutrophilic diseases. Dermatol. Clin..

[48] Shao S (2019). Neutrophil extracellular traps promote inflammatory responses in psoriasis via activating epidermal TLR4/IL-36R crosstalk. Front. Immunol..

[49] Gitlin AD (2020). Integration of innate immune signalling by caspase-8 cleavage of N4BP1. Nature.

