# Supplementary material for: The endoribonuclease N4BP1 prevents psoriasis by controlling both keratinocytes proliferation and neutrophil infiltration
Source: Cell Death Dis. 2021 May 14;12(5):488. doi: 10.1038/s41419-021-03774-w (PMC8121926; doi:10.1038/s41419-021-03774-w)
Supplement: Supplementary file 1 — Supplemental Figure Legends [file 41419_2021_3774_MOESM1_ESM.docx]

**The endoribonuclease N4BP1 prevents psoriasis by controlling both keratinocytes proliferation and neutrophil infiltration**

Chenliang Gou ^1,2, #^, Wenkai Ni ^3, #^, Panpan Ma ^1, 2, #^, Fengbo Zhao ^4, 5, #^, Zhou Wang ^5, 6^, Rong Sun ^4^, Yingcheng Wu ^1,5^, Yuanyuan Wu ^4^, Miaomiao Chen ^4^, Hao Chen ^3^, Jie Zhang ^2^, Yu Shen ^7^, Mingbin Xiao ^3^, Cuihua Lu ^3, *^, Renfang Mao ^5, *^, Yihui Fan ^1, 2, 4, *^

**Supplemental Figure Legends**

**Figure S1 Establishment of N4BP1 knockout mice.**

(A) The CRISPR-CAS9 mediated deletion of the second exon of mouse N4BP1 gene. The location of sgRNAs and the primers for genotyping was shown. (B) Genotyping was performed by genomic PCR to indicate the deletion of the second exon of mouse N4BP1 gene. (C) Representative photos of IMQ-induced psoriatic skin from ears of N4BP1 wildtype and knockout mice. The images (B, C) show representative data from one of three independent experiments.

**Figure S2 The role of N4BP1 in keratinocytes.**

(A) The up-regulated genes encoding keratin71 (KRT71) was further examined in skin from N4BP1 wildtype and knockout mice by RT-PCR. (B) Keratinocytes were isolated from neonatal N4BP1 wildtype and knockout mice skin. Representative images were shown and the N4BP1 knockout keratinocytes proliferate more quickly compared to wildtype keratinocytes.

**Figure S3 CRISPR-Cas9 mediated N4BP1 knockout enhances the expression of JunB and FosB.**

(A, B) The protein level of N4BP1, JunB and FosB was examined by WB in HeLa cells that stably transfected with Flag-N4BP1 and control vector. (C) N4BP1 stable knockout cells were established in HEK293T cells. The protein level of N4BP1 was checked by WB. (D) The mRNA level of JunB and FosB was determined by real-time RT-PCR in N4BP1 knockout 293T cells. (E) The protein level of JunB and FosB was examined by WB in N4BP1 knockout 293T cells. (F) The control and N4BP1 knockout 293T cells were treated with actinomycin (20uM) for indicated time and the mRNA level of JunB and FosB were examined by real-time RT-PCR at different time point. The images (A, B, C, E) show representative data from one of three independent experiments. Data (D, F) from one of three experiments are shown. Statistical differences between groups were determined by the Student’s t test. *, P < 0.05; **, P < 0.01; ***, P < 0.001

**Figure S4 R848 induces faster proliferation of N4BP1 knockout keratinocytes compared to normal controls.**

(A) N4BP1 wildtype and knockout keratinocytes were isolated and labeled with CFSE fluorescence. The CFSE labeled cells were further cultured in the presence of R848 (10uM) for indicated dates and the fluorescence was captured by fluorescence microscopy. (B) The CFSE labeled N4BP1 wildtype and knockout keratinocytes were cultured *in vitro* in the presence of R848 for five days and the fluorescence was examined by FACS. The images show representative data from one of three independent experiments.

**Figure S5** A model to demonstrate the role of N4BP1 in control of keratinocytes and neutrophils. N4BP1 acts as endoribonuclease to bind and degrade the mRNAs encoding JunB, FosB and CXCL1.

**Table S1 List of primers**

| Name | Oligo sequences (5’ – 3’) |
| --- | --- |
| Human/Mouse 18S, forward primer: | gaacgagactctggcatgcta |
| Human/Mouse 18S, reverse primer: | cacgctgagccagtcagtgta |
| Human JUNB, forward primer: | CAGGAGGGCTTCGCCGACGGC |
| Human JUNB, reverse primer | AGTAGCTGCTGAGGTTGGTGT |
| Human JUNC, forward primer: | GAACGTGACAGATGAGCAGGA |
| Human JUNC, reverse primer: | CCGGCGGCTCGCTGTGCAGGC |
| Human FOSB, forward primer: | GAGGAGAAGCGAAGGGTGCGC |
| Human FOSB, reverse primer: | CCCGGTTTGTGGGCCACCAGC |
| Human FOSC, forward primer: | AGCTGACTGATACACTCCAAG |
| Human FOSC, reverse primer: | CAGGCAGGTCGGTGAGCTGCC |
| Human CXCL1, forward primer: | CAAAGTGTGAACGTGAAGTCCC |
| Human CXCL1, reverse primer: | GCTTTCCGCCCATTCTTGAG |
| Human CCL20, forward primer: | GGCGAATCAGAAGCAGCAAG |
| Human CCL20, reverse primer: | AGCATTGATGTCACAGCCTT |
| Human S100A8, forward primer: | TGTCTCTTGTCAGCTGTCTT |
| Human S100A8, reverse primer: | ACTTGTGGTAGACGTCGATG |
| Human TNF-α, forward primer: | TCTGGGCAGGTCTACTTTGG |
| Human TNF-α, reverse primer: | TGAGCCAGAAGAGGTTGAGG |
| Human IL-17, forward primer: | AGCAGGCACAAACTCATCCA |
| Human IL-17, reverse primer: | TTGGGCATCCTGGATTTCGT |
| Mouse JUNB, forward primer: | TCACGACGACTCTTACGCAG |
| Mouse JUNB, reverse primer: | CCTTGAGACCCCGATAGGGA |
| Mouse JUNC, forward primer: | CCTTCTACGACGATGCCCTC |
| Mouse JUNC, reverse primer: | GGTTCAAGGTCATGCTCTGTTT |
| Mouse FOSB, forward primer: | TTTTCCCGGAGACTACGACTC |
| Mouse FOSB, reverse primer: | GTGATTGCGGTGACCGTTG |
| Mouse FOSC, forward primer: | CGGGTTTCAACGCCGACTA |
| Mouse FOSC, reverse primer: | TTGGCACTAGAGACGGACAGA |
| Mouse CXCL1, forward primer: | CCTATCGCCAATGAGCTGC |
| Mouse CXCL1, reverse primer: | CTCGCGACCATTCTTGAGTG |
| Mouse CCL20, forward primer: | CGTCTGCTCTTCCTTGCTTT |
| Mouse CCL20, reverse primer: | ACAGTCGTAGTTGCTTGCTG |
| Mouse S100A8, forward primer: | GTTGTCTCCATAGCCCGAGG |
| Mouse S100A8, reverse primer: | TCCAGTTCAGACGGCATTGTC |
| Mouse S100A9, forward primer: | ACTGGGCTTACACTGCTCTT |
| Mouse S100A9, reverse primer: | AGGTGTCGATGATGGTGGTT |
| Mouse CD45, forward primer: | GTGTAACTCCTACTAGCTACT |
| Mouse CD45, reverse primer: | GCAGGACAATGGTGACCACAC |
| Mouse CD4, forward primer: | GCTCTGCATCCTCTGCTGTGT |
| Mouse CD4, reverse primer: | GATTATGGCTCTTCTGCATCC |
| Mouse CD8, forward primer: | GGAGCCGAAAGCGTGTTTGCA |
| Mouse CD8, reverse primer: | CATGTAGTAGTTGTAGCTTCC |
| Mouse CD11B, forward primer: | GCTTGTGAGCAGCACTGAGAT |
| Mouse CD11B, reverse primer: | TCATCATGTCCTTGTACTGCC |
| Mouse CD11C, forward primer: | GGTCTGCTGCTGCTGGCTATC |
| Mouse CD11C, reverse primer: | CCAACATCTCCTTGTACTGAC |
| Mouse LY6G, forward primer: | GCGTTGCTCTGGAGATAGAAG |
| Mouse LY6G, reverse primer: | CCAGTGATCTCAGTATTGTCC |
| Mouse F4/80, forward primer: | GTCATCTATGCCATCCACTTC |
| Mouse F4/80, reverse primer: | ACAATCTTCAGACATTGTTCC |
| Mouse KRT1, forward primer: | CTGTCTGTTCCCCTAGTGGC |
| Mouse KRT1, reverse primer: | GTCCGGGTTGTGGTGTCTAC |
| Mouse KRT10, forward primer: | CTGGCGATGTGAACGTGGAA |
| Mouse KRT10, reverse primer: | GTCCCTGAACAGTGCGTCTC |
| Mouse KRT34, forward primer: | CTGTGTGCTAAGTCTGAGAACG |
| Mouse KRT34, reverse primer: | GCTGCCTCATGGACAGCTC |
| Mouse KRT71, forward primer: | ATGAGCCGCCAATTCACCTG |
| Mouse KRT71, reverse primer: | CTGCCCGGTAGGAGGATGA |
| Mouse KRT83, forward primer: | AGTGTTTGGAACTGGAAACTTCA |
| Mouse KRT83, reverse primer: | GCCTCAGCGTCTCGATGTA |
|  |  |
|  |  |

**Table S2 List of antibodies**

| **Antibodies** | **Source** | **Identifier (**Cat#) |
| --- | --- | --- |
| Anti-N4BP1 | Abcam | ab197079 |
| Anti-FLAG | Sigma | F3165 |
| Anti-MPO | ABclonal | A1374 |
| Anti-JUNB | Affinity Biosciences | AF6198 |
| Anti-FOSB | Affinity Biosciences | AF5010 |
| Anti-HSP90 | Santa Cruz | SC13119 |
| Anti-HSP70 | Santa Cruz | SC32239 |
| Anti-Mouse CD45.2-FITC | Elabscience | E-AB-F1122UC |
| Anti-Mouse LY6G-PE | Elabscience | E-AB-F1108UD |
| Anti-Mouse/Human CD11B-APC | Elabscience | E-AB-F1081UE |
| Anti-ANNEXIN-V-APC | Elabscience | E-CK-A117 |
| Cell Trace™ Cell Proliferation Kits | Thermo Fisher | C34570 |
|  |  |  |
