# Supplementary figures and images for: The endoribonuclease N4BP1 prevents psoriasis by controlling both keratinocytes proliferation and neutrophil infiltration
Source: Cell Death Dis. 2021 May 14;12(5):488. doi: 10.1038/s41419-021-03774-w (PMC8121926; doi:10.1038/s41419-021-03774-w)

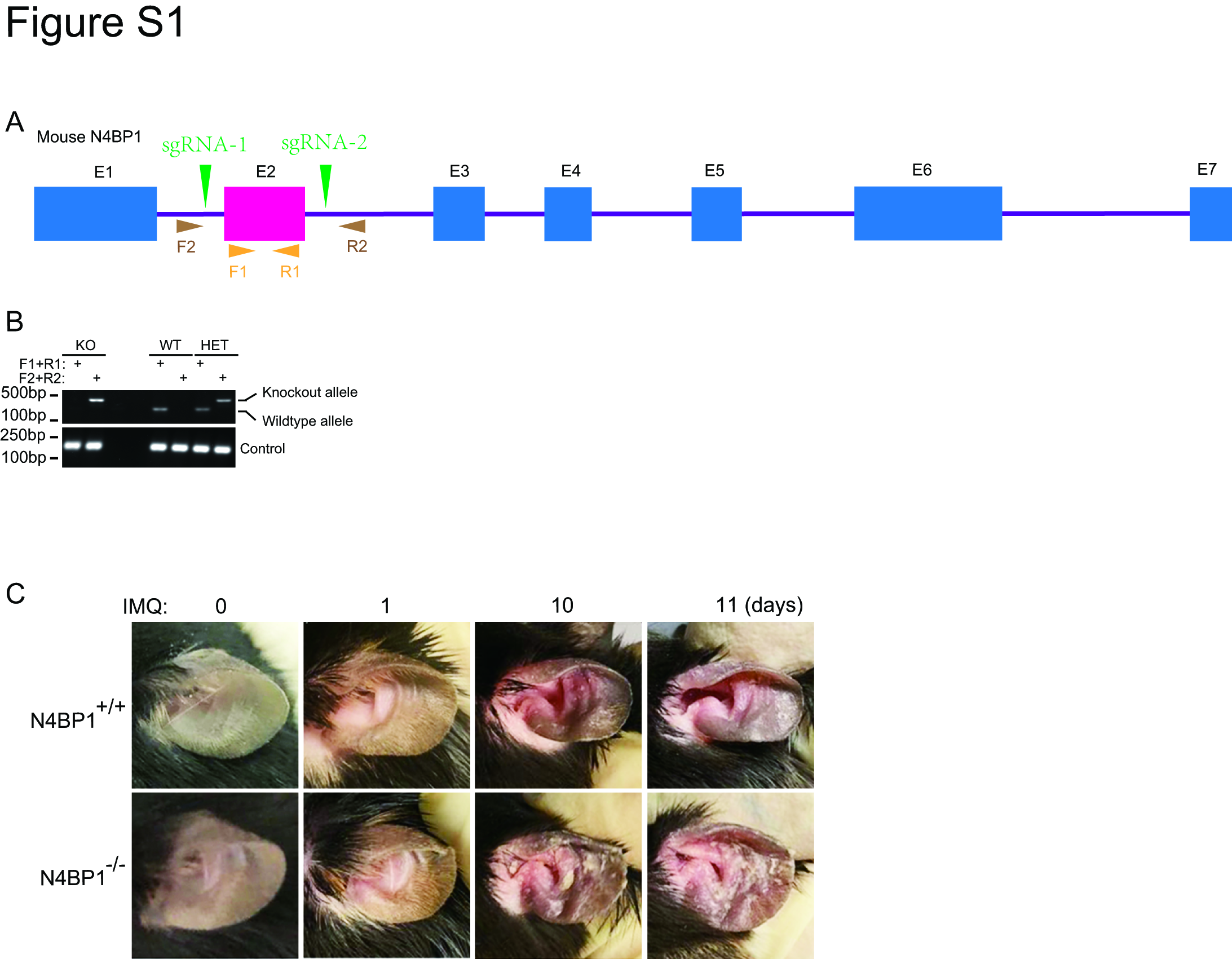

Supplement: Supplementary file 2 — Supplemental Figure S1 [file 41419_2021_3774_MOESM2_ESM.tif]

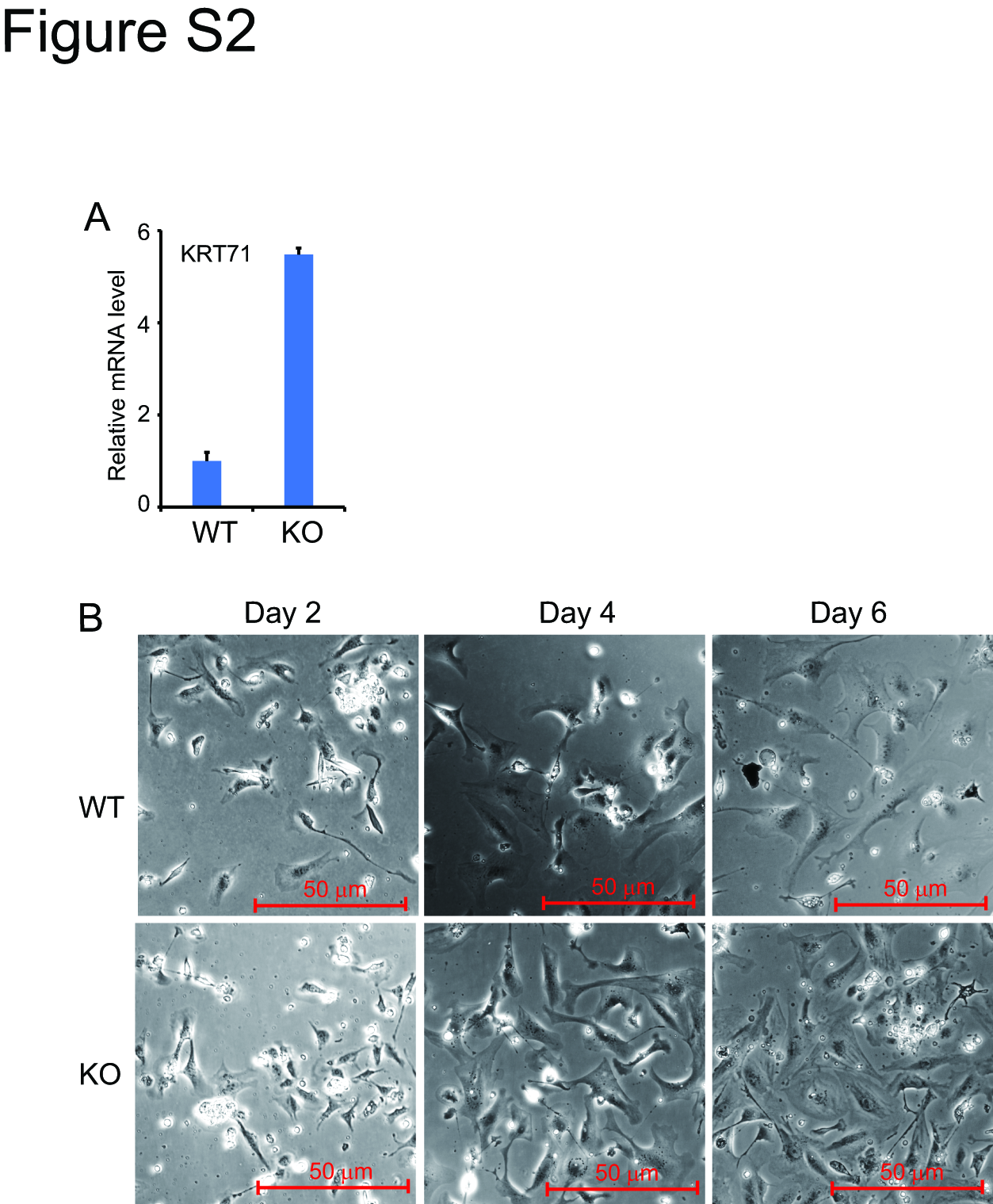

Supplement: Supplementary file 3 — Supplemental Figure S2 [file 41419_2021_3774_MOESM3_ESM.tif]

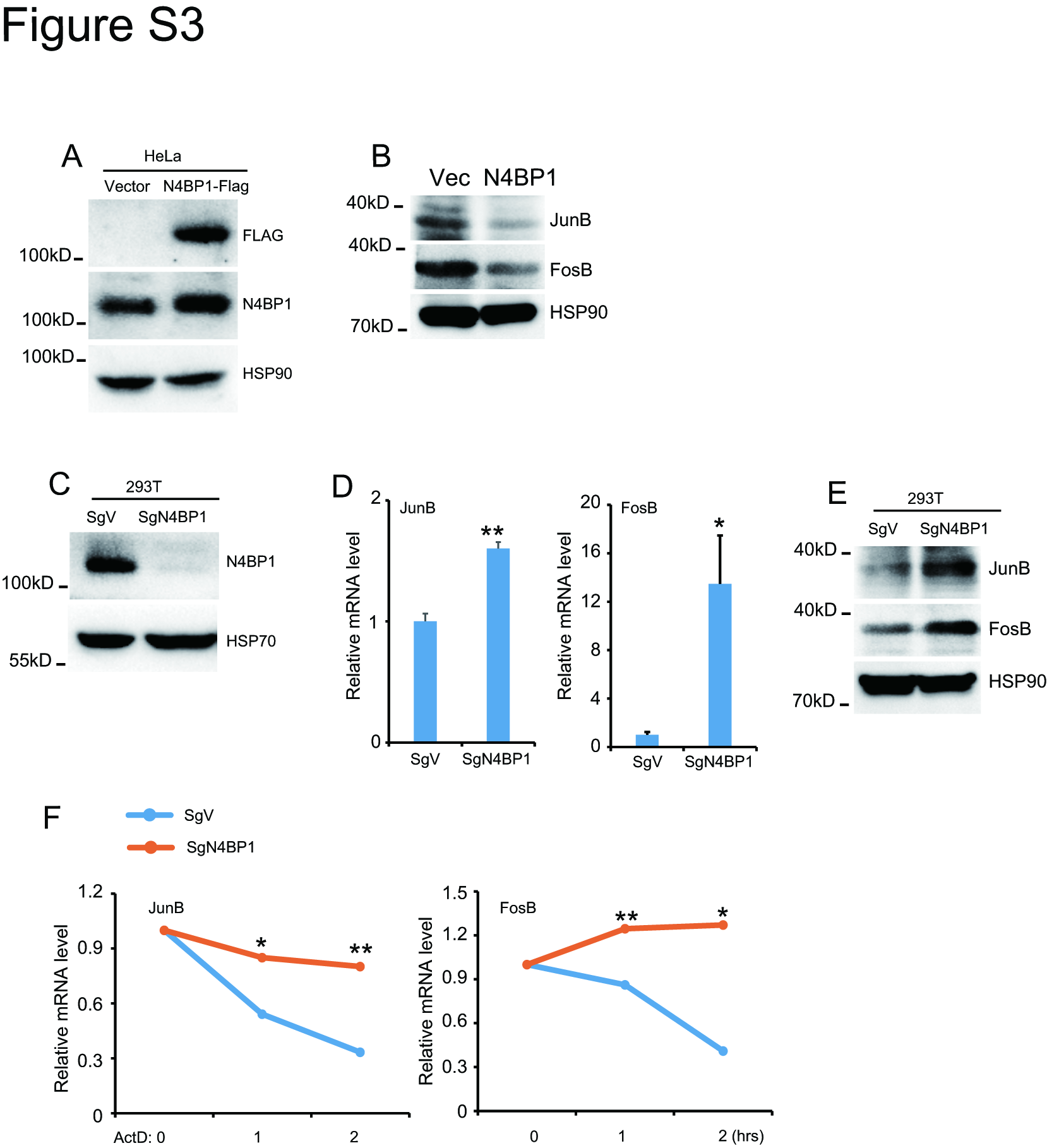

Supplement: Supplementary file 4 — Supplemental Figure S3 [file 41419_2021_3774_MOESM4_ESM.tif]

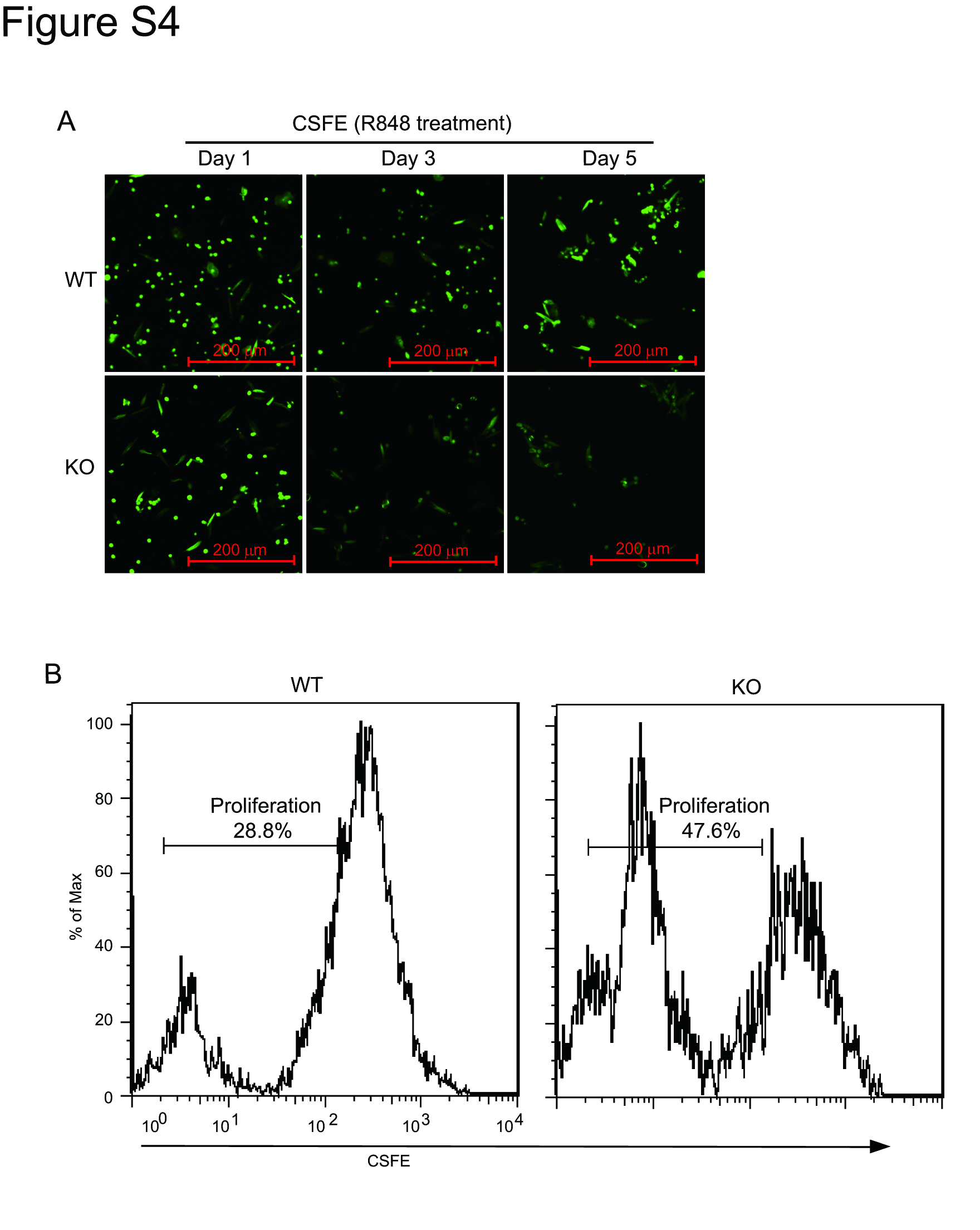

Supplement: Supplementary file 5 — Supplemental Figure S4 [file 41419_2021_3774_MOESM5_ESM.tif]

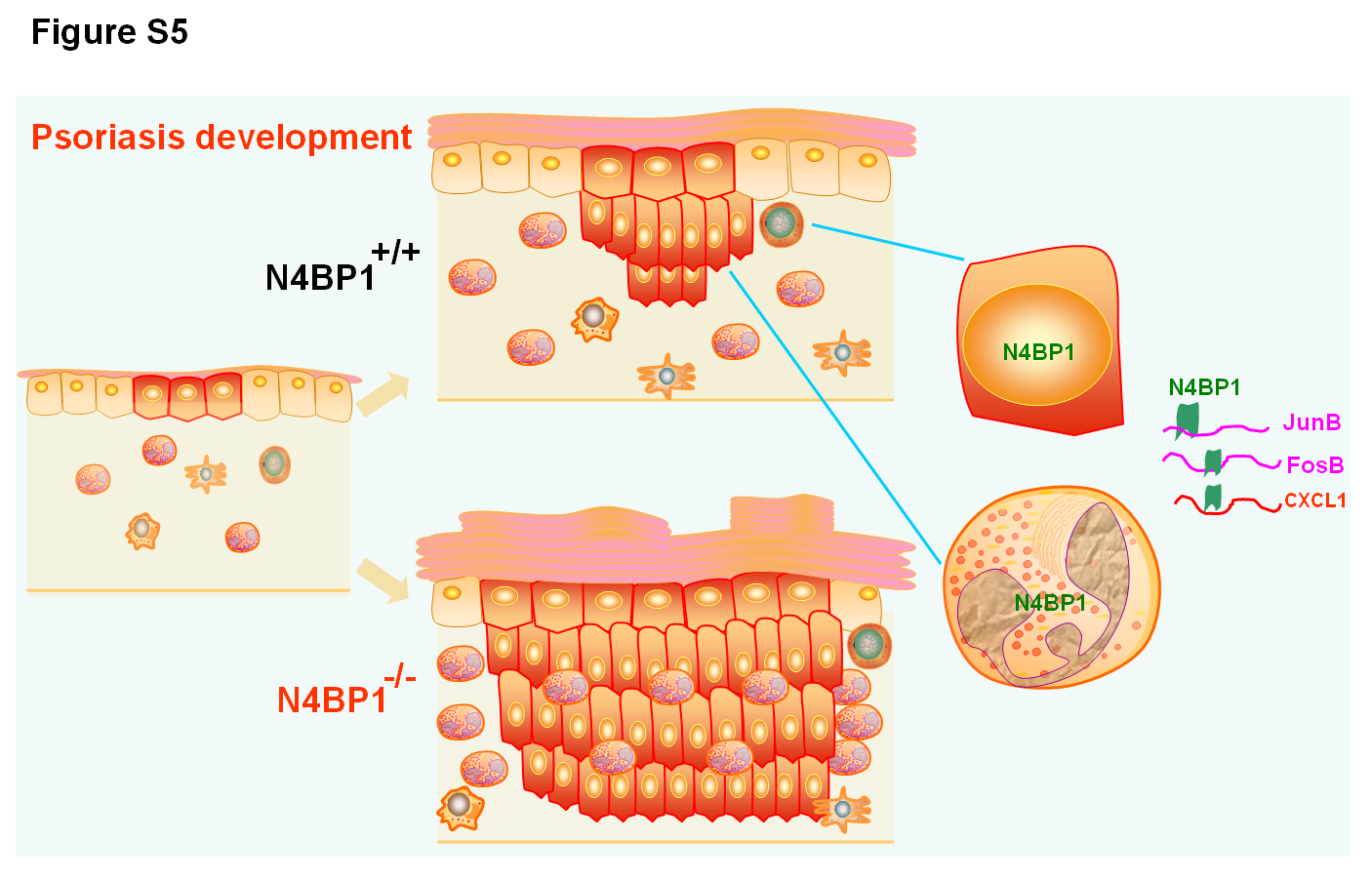

Supplement: Supplementary file 6 — Supplemental Figure S5 [file 41419_2021_3774_MOESM6_ESM.tif]
